# Plasma thrombomodulin levels are associated with acute kidney injury in patients with acute heart failure

**DOI:** 10.1080/07853890.2022.2142660

**Published:** 2022-11-10

**Authors:** Shu-Min Lin, Chih-Hsiang Chang, Ting-Yu Lin, Allen Chung-Cheng Huang, Chiung-Hung Lin, Yung-Chang Chen, Pao-Hsien Chu

**Affiliations:** a Department of Thoracic Medicine, School of Medicine, Chang Gung Memorial Hospital, Chang Gung University, Taipei, Taiwan; b Department of Respiratory Therapy, School of Medicine, Chang Gung Memorial Hospital, Chang Gung University, Taipei, Taiwan; c Department of Nephrology, School of Medicine, Chang Gung Memorial Hospital, Chang Gung University, Taipei, Taiwan; d Department of Cardiology, School of Medicine, Chang Gung Memorial Hospital, Chang Gung University, Taipei, Taiwan

**Keywords:** Cardiorenal syndrome, heart failure, endothelial dysfunction, kidney injury

## Abstract

Cardiorenal syndrome type I (CRS I) is defined as the development of acute kidney injury (AKI) following acute decompensated heart failure (ADHF). The clinical significance of endothelial markers in ADHF-associated AKI has yet to be clarified. This study therefore investigated the biological processes linking ADHF and AKI with the aim of determining whether the plasma markers of endothelial injury and activation are associated with AKI in patients with ADHF. The study prospectively recruited 125 consecutive patients admitted to a coronary critical unit due to ADHF. Patients with and without AKI were compared in terms of soluble thrombomodulin (sTM), angiopoietin (Ang)-1 and −2 plasma levels as well as baseline characteristics. Among the study population, 14 (11.2%) patients developed CRS within 7 days after admission. The hemoglobin levels (median [IQR]11.3[10.8–12.6] vs. 13.5 [12.2–15.0] g/dL, *p* = 0.003) and baseline eGFR (66.5[35.7–87.9] vs. 78.5 [64.9–107.5] mL/minute/1.73m^2^, *p* = 0.044) of patients with CRS were lower than those of patients without CRS. Patients with CRS also presented elevated plasma levels of BNP (1317.5 [222.6–3375.5] vs. 258.2 [63.2–925.8] pg/mL, *p* = 0.008), Ang-2 (3993.0 [1561.3–15722.7] vs. 1805.9 [1196.9–3302.3] pg/mL, *p* = 0.006), and sTM (6665.7 [4707.1–11947.3] vs. 4132.2 [3338.0–5531.8] ng/mL, *p* < 0.001), compared to patients without CRS. Multivariate logistic regression analysis based on forward stepwise method identified that log sTM was the only independent risk factor for AKI (OR, 13.83; 3.02–63.28, *p* = 0.001). Furthermore, higher sTM levels were associated with AKI in patients with ADHF. These findings suggest a novel approach to dealing with kidney injury in the context of ADHF, involving the use of baseline biomarker profiles to identify individuals at risk of developing AKI.

**KEY MESSAGES**
- The clinical significance of endothelial markers in acute decompensated heart failure (ADHF)-associated acute kidney injury (AKI) has not previously been clarified. This study revealed that markers of endothelial injury (i.e. plasma soluble thrombomodulin (sTM) levels) were higher in ADHF patients with AKI than in those without AKI.
- Multivariate analysis identified sTM level > cutoff value of 4,855.2 pg/mL as an independent factor associated with the development of AKI. sTM could potentially be used as a biomarker to predict the development of AKI in patients with heart failure.
- These findings suggest a novel approach to dealing with kidney injury in the context of ADHF, involving the use of baseline biomarker profiles to identify individuals at risk of developing AKI.

## Introduction

There is emerging evidence indicating a link between heart failure and renal insufficiency. The occurrence of cardiac and renal dysfunction can have a profound impact on clinical outcomes [1,2]. Cardiorenal syndromes are disorders of the heart and kidneys, whereby dysfunction in one organ leads to dysfunction in the other organ. Cardiorenal syndrome type I (CRS I) is defined as the development of acute kidney injury (AKI) following acute decompensated heart failure (ADHF) [3]. According to previous reports, AKI events developed in 12–37% of patients hospitalized for ADHF [4]. Patients with CRS I may have worse clinical outcomes including increased cardiovascular mortality rate, longer length of hospital stays, and higher chances of re-hospitalization [5].

The complex pathophysiology of CRS type 1 has yet to be fully elucidated. Recent evidence suggests that endothelial activation/injury are involved in pathogenesis of CRS I [6]. Endothelial injury may cause decreased glomerular filtration rates and impaired diuretic efficacy. These effects subsequently result in fluid retention and venous congestion, therefore, the cardiac output and renal perfusion are depressed [7]. Thrombomodulin (TM) and angiopoietins (Ang) play important roles in the process of endothelial activation and injury [8,9]. TM, a membrane-bound glycoprotein predominantly expressed in endothelial cells, has been shown to neutralize the clotting activity of thrombin and activate the anti-coagulant and anti-inflammatory properties of protein C [10]. A circulating soluble form of TM (sTM) is also released into the plasma through proteolytic degradation. The fact that sTM is released from the surfaces of endothelial cells only after injury makes it a recognizable marker of endothelial injury [11]. One previous study reported elevated sTM levels in patients with acute heart failure but not in those with chronic heart failure [12]. Researchers have also reported that elevated sTM plasma levels are associated with kidney injury following sepsis [8] and acute myocardial infarction [13].

The site to which Ang-1 and Ang-2 bind is the same as that of the endothelial cell-specific Tie-2 receptor [14]. Ang-1 and Ang-2 are crucial to vascular development, maturation, and inflammation. After binding to the Tie-2 receptor, Ang-1 triggers the activation of Tie-2, whereas Ang-2 exerts an antagonistic response to Tie-2. Ang-1 is protective in its anti-inflammatory effects and stabilization of the endothelium, whereas Ang-2 promotes an inflammatory response by activating endothelial cells and enhancing vascular leakage [15]. The importance of angiopoietins in endothelial activation and injury to the vascular barrier has prompted extensive research into their role as biomarkers in critical illness [16]. Plasma Ang-2 levels are significantly higher in cases of acute heart failure than in healthy controls, making them a predictor of poor outcomes [17]. They are also an independent predictor of mortality in patients undergoing dialysis in intensive care units [18].

The markers of endothelial injury and dysfunction have been associated with poor outcomes in patients with cardiovascular disease; however, the clinical role of endothelial injury markers in predicting AKI events of heart failure patients has not been clarified. This study investigated the underlying biological processes linking ADHF and AKI, with the aim of determining whether the plasma markers of endothelial injury and activation are associated with AKI in patients with ADHF.

## Materials and methods

### Study population

This study was conducted between November 2012 and September 2013 in the 14-bed coronary care unit (CCU) of a tertiary medical center (Chang Gung Memorial Hospital). The study prospectively recruited 125 consecutive patients who were admitted to the CCU due to ADHF. The reasons for CCU admission included: acute coronary syndrome, ADHF, or impending HF with the need for intensive monitoring. ADHF was diagnosed in accordance with the European Society of Cardiology criteria [19]. Exclusion criteria included age under 18 years old, having undergone organ transplant prior to admission, or end-stage renal failure. Plasma specimens were obtained from all patients within 24 h of CCU admission, and all patients were followed until hospital discharge or death. The Institutional Review Board of Chang Gung Memorial Hospital approved the protocols for recruitment and sample collection. Informed written consent was obtained from all participants.

### Data collection

The study collected the demographic data of patients on the first day of admission to the CCU. The underlying comorbidities were recorded. The left ventricular ejection fraction (LVEF) was derived using the modified Simpson’s method via echocardiography at the time of admission. The major adverse cardiac events (MACE) were defined as patients with previous history of recurrent myocardial infarction (MI), hospital admission for heart failure, unplanned repeat revascularization, malignant dysrhythmia, stroke, and pulmonary embolism [16].

The estimated glomerular filtration rate (eGFR) was calculated using the Chronic Kidney Disease Epidemiology Collaboration (CKD-EPI) equation [20]. Cases of baseline eGFR <60 were defined as chronic kidney disease (CKD). High-sensitivity C-reactive protein (hsCRP), hemoglobin (Hb), platelet, and white blood cell (WBC) counts were measured upon admission. The blood samples were collected into EDTA vacutainers and placed on ice immediately. Within 30 min of collection, samples were centrifuged at 3000 rpm (1000 g) and 4 °C for 15 min. Plasma was kept frozen in aliquots. Complete blood cell count and all biochemical analyses were performed in the Department of Laboratory Medicine, Chang Gung Memorial Hospital. Our hospital authority has permitted the checking of serum BNP levels in all patients with heart failure since January 2006. Serum BNP levels were measured using the microparticle enzyme immunoassay test (Abbott, Chicago, IL, USA). In this study, all BNP levels were obtained within one hour after presentation at the emergency department or ICU. The low osmolar iodine contrast was used when imaging examination was needed.

### Outcome assessment

The dependent variable in this study was the occurrence of acute CRS 1. AKI events were defined as any of the following criteria occurring within 7 days after admission: an increase in serum creatinine levels of **≥**0.3 mg/dL within a period of 48 h or an increase in serum creatinine level of **≥**1.5 times from baseline within a period of 7 days. Note that both of these criteria are recommended in the Kidney Disease Improving Global Outcomes practical clinical guidelines for AKI [21].

## Enzyme-linked immunosorbent assay (ELISA)

Plasma was collected from blood taken at the time of admission. The concentrations of sTM, von Willebrand factor (vWF), Ang-1, Ang-2, and Tie-2 were determined by ELISA in accordance with the manufacturer’s instructions (Sekisui Diagnostics, MA). The measured values were compared between patients with and without AKI.

## Statistical analysis

All data are expressed as medians with interquartile ranges (IQR) or mean ± standard deviation (S.D.) unless otherwise indicated. Equality of variances between groups was be assessed by Levene’s test. An unpaired Student’s *t*-test was used for normally distributed quantitative variables, while the Mann–Whitney test was used for non-normally distributed variables. Categorical variables were tested using the Chi-square test or Fisher’s exact test. Receiver operating characteristic (ROC) curves for day 1 plasma levels of Ang-2 and sTM were plotted to predict the development of AKI, and the respective areas under the ROC curves (AUROC) were calculated. Cut-off values were derived by the ROC curves using threshold values maximizing the sum of sensitivity and specificity. Univariate analysis was primarily performed to search for predictors for AKI. After identifying the variables with a *p*-value **<**0.1 in univariate regression analysis, a multivariate logistic regression analysis (stepwise forward) was used to determine independent predictive factors for the development of AKI. A *p*-value <0.05 using a two-sided test was considered statistically significant. All analysis was performed using SPSS software package, version 20.0 (SPSS, Inc, Chicago, IL, USA).

## Results

This study recruited a total of 125 consecutive patients. Among them, 14 (11.2%) patients developed CRS within 7 days after admission to the CCU. The demographic characteristics, biomarkers, and clinical presentations of this group are listed in Table 1. Compared with patients without CRS, those with CRS were older (median [IQR], 73[66.8–75.8]) vs.63 [54–74] years old; *p* = 0.024), had a lower incidence of coronary heart disease (35.7% vs. 71.2%, *p* = 0.013), were less likely to use ACEI/ARB (57.1 vs. 84.7%, *p* = 0.022), and were more likely to have LVEF <40% (50.0 vs. 22.5%, *p =* 0.026). Compared to patients without CRS, those with CRS presented lower median hemoglobin levels (11.3[10.8–12.6] vs. 13.5 [12.2–15.0] g/dL, *p* = 0.003) and baseline eGFR levels (66.5[35.7–87.9] vs. 78.5 [64.9–107.5] mL/minute/1.73m^2^, *p* = 0.048). Patients with CRS also presented higher BNP median plasma levels (1317.5 [222.6–3375.5] vs. 258.2 [63.2–925.8] pg/mL, *p* = 0.008), Ang-2 (3993.0 [1561.3–15722.7] vs. 1805.9 [1196.9–3302.3] pg/mL, *p* = 0.006), and sTM (6665.7 [4707.1–11947.3] vs. 4132.2 [3338.0–5531.8] ng/mL, *p* < 0.001). No significant differences were observed between the two groups in terms of gender, comorbidities, CKD, smoking status, body mass index (BMI), blood pressure, heart rate, leukocyte count, hsCRP, VEGF, vWF, Tie-2, or Ang-1 levels. The 90 days mortality rate was 7.1% (1/14) and 5.4% (6/111) in patients with and without AKI, respectively. Among the cohort, there was 1 patient developed end-stage kidney disease in 1 year period.

**Table 1.** Baseline characteristics of patients with acute decompensated heart failure.

	Entire cohort (*n* = 125)	CRS I (*n* = 14)	No CRS I (*n* = 111)	*p*-Value
Demographics				
Age, year	64 (55–74.5)	73 (66.8–75.8)	63 (54–74)	0.024
BMI	24.4 (22.3–26.7)	22.9 (21.0–27.0)	24.5 (22.5–26.6)	0.132
Male gender	92 (73.6)	8 (57.1%)	84 (75.7%)	0.195
Current smoker	56 (44.8%)	5 (35.7%)	51 (75.7%)	0.574
Previous heart failure	39 (31.2%)	4 (28.6%)	35 (31.5%)	1.00
Coronary heart disease	83 (66.4%)	5 (35.7%)	79 (71.2%)	0.013
Myocardial infarction	22 (17.6%)	3 (21.4%)	19 (17.1%)	0.712
Hypertension	80 (64.0%)	12 (85.7%)	68 (61.3%)	0.084
Diabetes	52 (41.6%)	6 (42.9%)	46 (41.4%)	1.00
Chronic atrial fibrillation	14 (11.2%)	2 (14.3%)	12 (10.8%)	0.657
Chronic kidney disease	26 (20.8%)	5 (35.7%)	21 (18.9%)	0.166
MACE	28 (22.2%)	6 (42.8%)	22 (19.8%)	0.083
Clinical presentation				
NYHA class III/IV	45 (36.0%)	5 (35.7%)	40 (36.0%)	1.00
Systolic BP, mmHg	125 (111.0–142.5)	125 (114–154)	125 (111–142)	0.519
Diastolic BP, mmHg	72 (64–82)	71 (58.8–83.0)	72 (65–82)	0.556
Heart rate, beat/min	80 (68–89.5)	76.5 (67–85.8)	80 (68–90)	0.352
LVEF < 40 %	32 (25.6%)	7 (50.0%)	25 (22.5%)	0.026
Medications				
Beta blocker	107 (85.6%)	12 (87.5%)	95 (85.6%)	1.00
ACEI/ARB	102 (81.6%)	8 (57.1%)	94 (84.7%)	0.022
Spirolactone	24 (19.2%)	2 (14.3%)	22 (19.8%)	1.00
Loop diuretics	52 (41.6%)	8 (57.1%)	44 (39.4%)	0.211
Clinical outcomes				
Length of stay, days	7 (6–10)	17 (9.8–19.5)	7 (5.8–9.3)	0.089
In-hospital mortality	7 (5.6%)	1 (7.1%)	6 (5.4%)	0.574
Laboratory parameters				
Leukocyte count, per mL	9600 (8100–11600)	10150 (8675–12450)	9350 (8025–11425)	0.272
Hemoglobin, g/dL	13 (11.6–14.8)	11.3 (10.8–12.6)	13.5 (12.2–15.0)	0.003
Sodium, mmol/L	139 (137–141)	139.5 (136.8–144.0)	139 (137–141)	0.287
Potassium, mEq/L	3.9 (3.6–4.2)	4.0 (3.5–4.8)	3.9 (3.7–4.2)	0.250
Creatinine, mg/dL	0.98 (0.77–1.19)	1.08 (0.86–1.54)	0.96 (0.77–1.19)	0.010
eGFR, ml/minute/1.73m^2^	77.9 (63.2–105.3)	66.5 (35.7–87.9)	78.5 (64.9–107.5)	0.044
hsCRP, mg/L	8.4 (2.6–34.1)	25.3 (4.0–48.7)	7.6 (2.6–29.1)	0.404
BNP, pg/mL	371.5 (65.5–997.7)	1317.5 (222.6–3375.5)	258.2 (63.2–925.8)	0.008
Troponin-I, ng/mL	0.7 (0.2–3.4)	0.5 (0.2–1.4)	0.7 (0.2–4.6)	0.576
Biomarkers of endothelial injury				
Thrombomodulin, pg/mL	4251.1 (3445.8–5847.9)	6665.7 (4707.1–11947.3)	4132.2 (3338–5531.8)	<0.0001
vWF, MU	667.2 (490.7–868.4)	541.4 (405.5–733.8)	675.4 (517.7–882.6)	0.129
Tie-2, ng/mL	7.6 (6.1–9.2)	7.0 (5.7–8.8)	7.7 (6.3–9.3)	0.334
Angiopoietin-1, pg/mL	23188.8 (12925–30153)	25849 (6394–30331)	22923 (13102–30064)	0.691
Angiopoietin-2, pg/mL	1892.4 (1236.6–3848.7)	3993 (1561.3–15722.7)	1805.9 (1196.9–3302.3)	0.006

CRS: cardiorenal syndrome; BMI: body mass index; MACE: major adverse cardiac events; NYHA: New York Heart association; BP: blood pressure; LVEF: left ventricular ejection fraction; ACEI: angiotensin-converting enzyme inhibitor; ARB: angiotensin II receptor antagonist; eGFR: estimated glomerular filtration rate; hsCRP: high-sensitive C-reactive protein; BNP: brain natriuretic peptide; vWF: von Willebrand factor.

**Figure 1.**
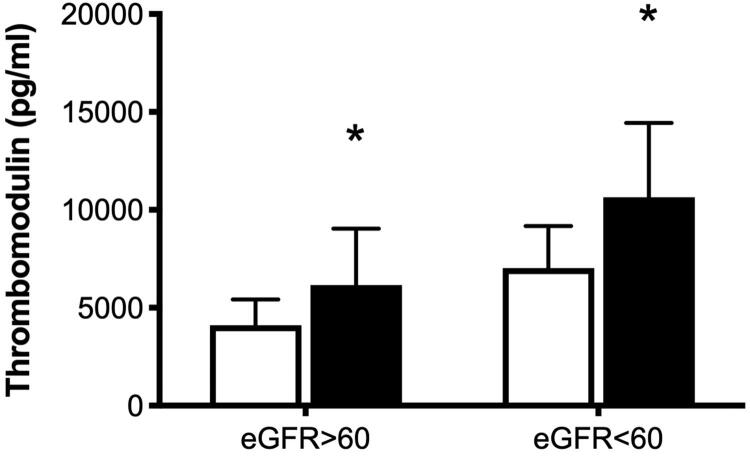
Concentrations of angiopoietin-2 (A) and thrombomodulin (B) in patients with and without acute kidney injury (AKI). Open bars, patients without AKI; solid bars, patients with AKI. *p < 0.05 compared to patients without AKI in the subgroup in which the estimated glomerular filtration rate (eGFR) was <60 ml/minute/1.73 m^2^. Data are expressed as mean ± standard deviation.

In the study population, 99 of the patients presented baseline eGFR > 60 mL/min/1.73m^2^, whereas 26 patients (with CKD) presented eGFR <60 mL/min/1.73m^2^. The incidence of CKD among patients with AKI (5/14, 35.7%) was similar to that among patients without CKD (21/111, 18.9%; OR, 2.38; 95% CI 0.72–7.84; *p* = 0.166). Among patients with eGFR < 60 mL/min/1.73m^2^, we observed higher Ang-2 plasma levels (11,573 ± 9,441 pg/mL, *n* = 5 vs. 4,652 ± 7,415 pg/mL, *n* = 21; *p* = 0.021) and sTM plasma levels (11,641 ± 3,795 pg/mL, *n* = 5 vs. 7,032 ± 2,147 ng/mL, *n* = 2; *p* = 0.008) in patients with AKI than in patients without AKI (Figure 1). Among patients with eGFR >60 mL/min/1.73m^2^, we also observed elevated Ang-2 plasma levels (5,883 ± 7,425 pg/mL, *n* = 9 vs. 2,951 ± 3,825 pg/mL, *n* = 90; *p* = 0.05) and sTM plasma levels (6,165 ± 2,874 pg/mL, *n* = 9 vs. 4,102 ± 1,323 pg/mL, *n* = 90; *p* = 0.0002) in AKI patients than in patients without AKI. This indicates that baseline eGFR levels were not associated with the difference between Ang-2 and sTM levels in patients with and without CRS.

Table 2 lists the ROC curves of Ang-2 and sTM used to predict development of CRS. AUROC revealed that Ang-2 and TM plasma levels had modest discriminative powers pertaining to the development of CRS (0.704, 95% CI 0.55–0.859, *p* = 0.013; and 0.789, 95% CI 0.675–0.903, *p* < 0.001, respectively). The cutoff values for Ang-2 and sTM in predicting AKI were 2,479 and 4,855.2 g/mL, respectively.

**Table 2.** Area under the receiver operating characteristic curves (AUROC) for day-1 plasma levels of angiopoietin-2 and thrombomodulin in predicting development of acute kidney injury.

	AUROC^a^	95% CI^b^	*p*-Value	Cutoff value	Sensitivity	Specificity
Angiopoietin-2	0.704	0.550–0.859	0.013	2,479.0 pg/mL	0.714	0.627
Thrombomodulin	0.789	0.675–0.903	<0.001	4,855.2 pg/mL	0.786	0.691

^a^AUROC: area under the receiver operating characteristic curve; ^b^ CI: confidence interval.

Table 3 lists the results of univariate and multivariate analysis of possible risk factors for AKI development in ADHF patients. Univariate regression analysis indicated that age, coronary heart disease, hypertension, MACE, LVEF, use of ACEI/ARB, levels of hemoglobin, BNP, sTM and Ang-2 were related to the development of AKI. After adjusting for the factors mentioned above using multivariate logistic regression analysis based on forward stepwise method, coronary heart disease and log sTM were selected for analysis. The log sTM was the only independent risk factor for AKI (OR, 13.83; 3.02–63.28, *p* = 0.001).

**Table 3.** Univariate and multivariate analysis of variables associated with acute kidney injury in heart failure patients.

	Univariate			Multivariate		
	OR	95% CI	*p*-Value	O.R.	95% CI	*p*-Value
Age, yrs	0.95	0.90–0.99	0.029			
Coronary heart disease	0.23	0.70–0.72	0.012	0.29	0.08–1.04	0.057
Hypertension	3.79	0.81–17.79	0.091			
MACE	0.33	0.10–1.05	0.060			
Log LVEF	0.23	0.06–0.92	0.037			
ACEI/ARB	0.24	0.07–0.78	0.018			
Hemoglobin	1.44	1.12–1.86	0.005			
Log BNP	0.63	0.36–1.08	0.093			
Log Thrombomodulin	15.67	3.62–67.82	<0.0001	13.83	3.02–63.28	0.001
Log Angiopoietin	2.28	1.29–4.03	0.004			

MACE: major adverse cardiac events; LVEF: left ventricular ejection fraction; ACEI: angiotensin-converting enzyme inhibitor; ARB: angiotensin II receptor antagonist; BNP: brain natriuretic peptide; OR: odds ratio; CI: confidence interval.

**Figure 2.**
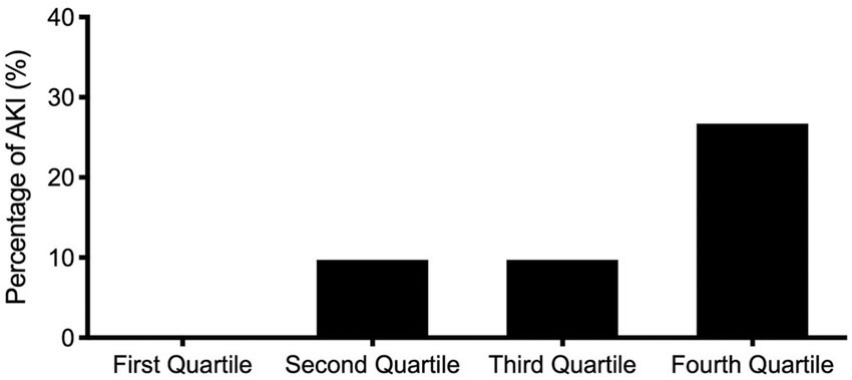
Percentage of acute kidney injury (y-axis) among subjects with acute decompensated heart failure stratified by quartiles of plasma thrombomodulin.

Figure 2 shows the incidence of AKI stratified by quartiles of sTM plasma levels. The incidence of AKI was markedly higher in the 4th quartile (8/30, 26.7%; *p* = 0.001) than in the other three quartiles. By contrast, the incidence of AKI was lower in the 1st quartile (0/32, 0%) than in the other three quartiles.

**Figure 3.**
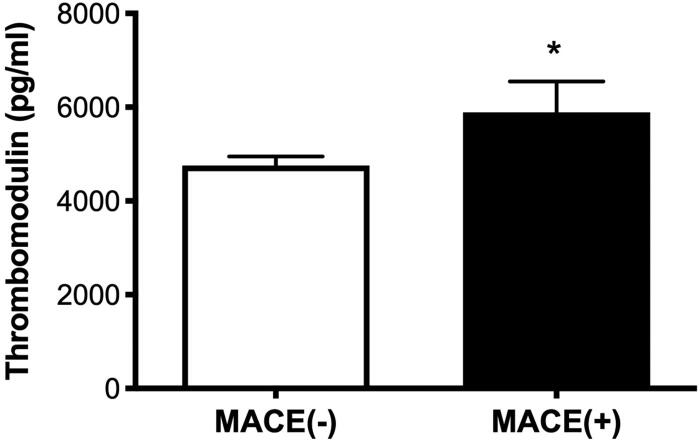
Plasma levels of thrombomodulin were significantly lower in patients without major adverse cardiac events (4,754.7 ± 1,896.8 pg/ml) than in patients with multivessel disease (5,892.1 ± 3,495.9 pg/ml, *p* = 0.026).

We also examined the correlations among sTM, Hb concentration, WBC count, hsCRP, and BNP levels. Table 4 lists the correlations among serum mediators and these variables. We observed a correlation between sTM levels and the concentrations of Hb (*r* = −0.516; *p* < 0.001) and BNP (*r* = 0.434; *p* = 0.001). sTM plasma levels were higher in patients with MACE than in those without MACE (5,892.1 ± 3,495.9 vs. 4,754.7 ± 1,896.8 pg/mL, *p* = 0.026) (Figure 3). In addition, there was a correlation between Ang-2 levels and eGFR (*r* = −0.270; *p* = 0.002).

**Table 4.** Correlation between levels of thrombomodulin and other markers.

	Hemoglobin	WBC count	hsCRP	LVEF	BNP
Thrombomodulin					
*R*	−0.516	−0.002	0.152	−0.059	0.434
*p*-Value	<0.001	0.978	0.182	0.517	0.001

WBC: white blood cell; hsCRP: high-sensitivity C-reactive protein; LVEF: left ventricular ejection fraction; BNP: brain natriuretic peptide.

## Discussion

The study showed that BNP, Ang-2, and sTM plasma levels were higher and Hb levels were lower in ADHF patients with AKI than in those without AKI. ROC curves revealed that day-1 Ang-2 and sTM levels had modest discriminative power in predicting the development of AKI. The difference between patients with and without CRS in terms of Ang-2 and sTM levels was unaffected by baseline renal function, as indicated by eGFR. Multivariate analysis identified sTM level was an independent factor associated with the development of AKI.

Our results revealed higher sTM plasma levels in ADHF patients with AKI [22]. In previous studies, sTM levels were identified as a specific marker of endothelial injury [11]. Our data suggest that endothelial injury may play a crucial role in mediating ADHF associated kidney injury. Heart failure associated altered hemodynamics, hypoxia, and inflammatory may be responsible for endothelial injury [6]. The procoagulatory processes caused by injured endothelial cells may promote microthrombotic foci formation leading to organ microcirculation failures or complete organ failure [23]. Elevated sTM levels have been reported in patients sepsis [8] and acute myocardial infarction [13] associated AKI. However, elevated sTM levels have never been reported in patients with CRS type I. Elevated sTM levels are related to several vascular-related diseases, including coronary disease, stroke, and peripheral occlusive arterial disease [24]. MACE has also been associated with poor prognosis in patients with AMI [16] or diabetes [25], and endothelial injury is reportedly involved in the development of MACE [26]. In the current study, we observed higher sTM levels in patients with MACE than in those without MACE, due perhaps to vascular atherosclerosis or endothelial injury of greater severity. As in one previous study [27], we found that LVEF <40% was associated with AKI. A decrease in ejection fraction reflects reduced cardiac output and renal hypoperfusion leading to functional AKI and parenchymal damage [28]. Endothelial injury is commonly observed in patients with CKD [29]. However, we found that the incidence of CKD in patients with AKI was similar to that in patients without AKI. The difference between patients with and without CRS in terms of Ang-2 and sTM levels was unaffected by baseline renal function, as indicated by eGFR. These results reflect that endothelial activation/injury is implicated in the acute episode of kidney injury in patients with ADHF. In addition, the elevated Ang2 and sTM levels in patients with ADHF were not caused by cardiorenal syndrome type 3 or 4.

As in previous studies, we determined that decreased hemoglobin levels and increased BNP levels are associated with CRS. Under cardiac stretch and ischemia, BNP is secreted by cardiomyocytes. Evidence suggests that BNP plays an important role in cardiorenal protection [30]. One recent study showed that ADHF patients with renal dysfunction had higher BNP levels than those with normal renal function [31]. In patients with heart failure, endothelial dysfunction may be caused by the decreased shear stress due to pump failure [32]. In addition to blood flow, shear stress was also affected by blood viscosity. Decreased blood viscosity due to a reduction in Hb concentration can result in depressed shear stress and subsequent ischemia-induced vasodilation [33]. We therefore postulate that anemia (common in cases of CRS) is a contributor to decreased shear stress, leading to degraded endothelial function and contributing to degraded cardiac and renal function [34]. This study also identified correlations among BNP, hemoglobin, and the markers of endothelial injury, as indicated by sTM plasma levels. These data provide evidence of a link between endothelial injuries induced by heart failure and subsequent kidney injury.

The results in this study demonstrate that sTM plasma levels early in the course of heart failure may be predictive of AKI development. The plasma levels of sTM could be used as a biomarker for the prediction of clinical outcomes or treatment decisions. The increased sTM plasma levels are associated with AKI development, which also suggests that endothelial injury may play a role in pathogenesis of type I CRS. Researchers are increasingly recognizing the clinical and biological heterogeneity of CRS. The pathophysiology of CRS represents the confluence of several hemodynamic, neuro-hormonal, inflammatory, and endothelial perturbations [35]. Our previous study had disclosed that elevated Ang-2 and sTM plasma levels were associated with development of AKI in patients with AMI [13]. In the present study, sTM was identified as an independent predictor for AKI in patients with heart failure. By identifying the markers of endothelial injury associated with AKI in different recruited subjects with various cardiovascular problems, these findings further emphasize the central role of endothelial injury in dysfunction of heart leading to kidney injury. Among mechanisms that link dysfunction of heart and kidney, endothelial injury seems to be one final common pathway. Incorporating novel biomarkers in combination with clinical predictors could greatly enhance the efficacy of diagnostic algorithms in predicting CRS [35]. These data may enhance our understanding of dysregulated coagulation and endothelial dysfunction as a pathogenic mechanism in CRS. The impact of endothelial dysfunction in AKI development may also be an important target for future research in heart failure patients.

This is the first study to report on the potential role of endothelial biomarkers for the prediction of AKI in ADHF patients. Note however that this study has a number of limitations that should be considered in interpreting the results. This observational study included only a small number of patients recruited from a single medical facility, which prevented the use of multivariate analysis. It is highly likely that baseline characteristics, such as illness severity, are linked to endothelial dysregulation as well as the risk of developing AKI. Furthermore, we were unable to establish a causal association between the endothelial dysfunction and AKI development based solely on these results. It will be necessary in the future to conduct larger-scale studies with adjustments for potential confounding factors. Further studies should also be conducted to evaluate the efficacy of monitoring plasma sTM levels for the prevention of AKI in patients with ADHF.

## Conclusions

In conclusion, this study revealed that higher sTM levels are associated with AKI in patients with ADHF. Subject to confirmation in large-scale prospective studies, our results suggest that ADHF patients with elevated sTM levels may require close monitoring of renal function or avoidance of nephrotoxic agents. Our current findings suggest a novel avenue by which to address kidney injury in the context of ADHF, wherein baseline biomarker profiles could potentially be used to identify individuals at risk of developing AKI.

## Data Availability

The datasets compiled in the current study are available from the corresponding author upon reasonable request.
